# Hyaluronic Acid as a Component of Natural Polymer Blends for Biomedical Applications: A Review

**DOI:** 10.3390/molecules25184035

**Published:** 2020-09-04

**Authors:** Alina Sionkowska, Magdalena Gadomska, Katarzyna Musiał, Jacek Piątek

**Affiliations:** 1 Department of Biomaterials and Cosmetics Chemistry, Faculty of Chemistry, Nicolaus Copernicus University in Toruń, Gagarin 7, 87-100 Toruń, Poland; 291013@stud.umk.pl (M.G.); musialk.97@gmail.com (K.M.); 2 Health Sciences Faculty, President Stanisław Wojciechowski State University of Applied Sciences in Kalisz, Nowy Świat 4 st., 62-800 Kalisz, Poland; drpiatek@interia.eu

**Keywords:** hyaluronic acid, polymer blends, biopolymers, biomaterials, chitosan, collagen

## Abstract

In this review, we provide a report on recent studies in the field of research on the blends of hyaluronic acid with other natural polymers, namely collagen and chitosan. Hyaluronic acid has attracted significant interest in biomedical and cosmetic applications due to its interesting properties. In recent years, blends of hyaluronic acid with other polymers have been studied for new materials development. New materials may show improved properties that are important in the biomedical applications and in cosmetic preparations. In this review paper, the structure, preparation, and properties of hyaluronic acid blends with collagen and chitosan have been discussed and examples of new materials based on such blends have been presented. A comparison of the currently available information in the field has been shown. Future aspects in the field of hyaluronic acid blends and their applications in the biomedical and cosmetic industry have also been mentioned.

## 1. Introduction

**Figure 1:**
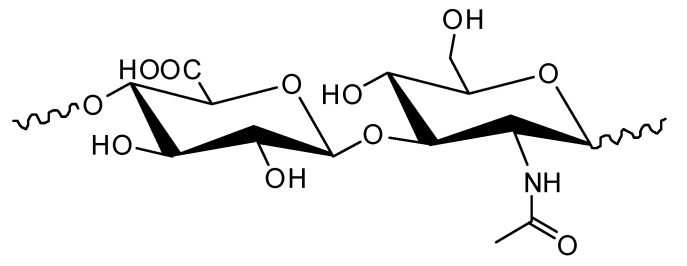
The structure of hyaluronic acid.

Hyaluronic acid (HA) belongs to the glycosaminoglycan compounds, which are members of the polysaccharides family. The molecule of HA is consisting of alternating units of *N*-acetyl-D-glucosamine and glucuronic acid. HA is a part of almost every tissue in vertebrates [1]. The molecular weight of HA is very high, up to several millions. The structure of HA is shown in Figure 1. HA is not covalently bonded to proteins, but it is widely distributed mainly in the connective tissue. Chemically, hyaluronic acid is a hydrophilic macromolecule with -COOH and -OH functional groups. The solubility of HA in water is high, and it forms highly viscous solutions. Such solutions show unique viscoelastic properties. HA can form intramolecular hydrogen bonding, which leads to three-dimensional structures. Hyaluronic acid can trap water within its structure and can form gels. The amount of trapped water is approximately 1000 times of the weight of HA. HA is a part of the following tissues: articular cartilage, the nucleus pulposus, skin, the cervix, and the glycocalyx of endothelial cells. The solutions and gels of HA are commonly used as a dermal filler. After injection, it is possible to restore skin volume and to minimize the appearance of wrinkles. HA is a very effective and safe ingredient of cosmetic formulation.

The role of hyaluronic acid in the body is strictly connected with its properties. HA is synthesized by a class of integral membrane proteins known as HA synthases [2]. These proteins lengthen HA by repeated addition of glucuronic acid and *N*-acetyl-d-glucosamine groups to the growing sugar. The half-life of HA in human tissues ranges from one day in skin tissues up to 70 days in some parts of the eye. HA plays an important role in ECM (extracellular matrix) by several specific and non-specific interactions. It is also helpful for the growth of epithelial tissue cells, eosinophil, macrophages, and a few animal tissues cells. The role usually depends on the molecular weight of HA. For example, low weight HA is essential in healing and scar formation, whereas high molecular weight HA may support the tissue integrity. HA due to water absorption provides flexibility to the animal tissue and lubrication in muscular connective tissues.

Hyaluronic acid is widely used in biomaterials. For example, HA has been extensively investigated for wound dressing applications. HA can be cross-linked by a variety of physical and chemical methods because of the presence of several functional groups. HA can be used in tissue repair because it is able to promote mesenchymal and epithelial cell migration and differentiation. Biological properties make HA very good material for tissue engineering. From an aqueous solution of HA, one can fabricate 3D porous materials. The properties of 3D HA materials, as well as the properties of HA hydrogels, can be modified by HA concentration and by crosslinking process [3,4,5,6,7]. The crosslinking process makes materials more resistant to enzymatic degradation. HA hydrogels are widely used for skin rejuvenation due to its ability to moisturize the skin [8]. Moreover, HA in intradermal injection has been used as a dermal filler. HA is also an ingredient in cosmetic creams and gels for the treatment of different types of diseases [9,10,11,12]. HA in the form of hydrogel works as a collagen stimulator [13,14,15]. The film-forming properties of HA can also be used for hair treatment [16].

Hyaluronic acid is widely used not only in cosmetics. HA is also used for drug delivery, coatings and implantation of organs, and for several therapeutic purposes due to its ability to modify cellular behavior. The properties of hyaluronic acid can be modified not only by chemical and physical crosslinking methods. Hyaluronic acid can be blended with another polymer and/or a biopolymer. Blending the polysaccharides with the less expensive synthetic polymers is one of the approaches to reduce the cost of materials. Polymer scaffolds for biomedical applications can be made of blends of hyaluronic acid with synthetic polymers and/or another natural polymer. The aim of this review is to show the current research on the blends of hyaluronic acid with collagen and chitosan. However, it should be emphasized that hyaluronic acid can also be blended with several other polymers and biopolymers [17,18,19,20].

## 2. Blends of Hyaluronic Acid and Collagen

Hyaluronic acid can be used for modification of collagen properties. Collagen is a structural protein that provides fundamental structural and mechanical support in human and animal bodies. Collagen-based materials are widely applied in the biomedical field, for example, it can be used in tissue regeneration/engineering and for new materials fabrication. The example of collagen application is a preparation of artificial skin, bone graft substitutes (collagen composites with inorganic particles), dental implants, artificial tendons and blood vessels, corneal implants, regeneration of nerves, cartilage, and several other tissues [21].

**Figure 2:**
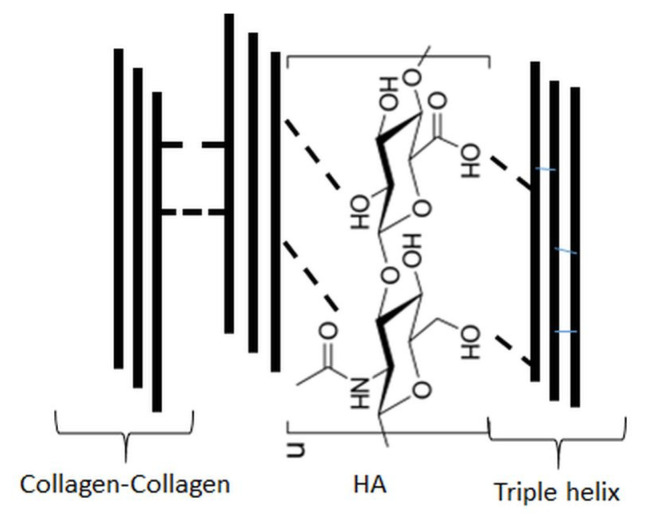
Possible interactions between collagen and hyaluronic acid (HA) in blends.

The investigation of the properties of the blends based on collagen and hyaluronic acid has been already initiated and several interesting biomaterials based on such blends have been proposed [22,23,24,25,26,27,28,29,30,31,32,33,34,35,36,37,38,39,40,41]. Several research groups have studied interactions between collagen and HA and the possibility of new materials formation based on such a blend. The possible interactions between collagen and hyaluronic acid are shown in Figure 2.

Polymer-polymer interactions, such as hydrophobic and electrostatic interactions, have been studied in a hyaluronic acid and collagen mixture, and new matrices based on the blends have been obtained by Taguchi et al. [22]. Matrices with polymer–polymer interactions could be obtained upon their immersion in water at 37 °C with no collagen denaturation. The material obtained showed high swelling properties [22]. Collagen–hyaluronic acid membranes for applications in regenerative medicine can be obtained by self-assembly [23]. The lyophilized matrices show multipore structures and can be useful in the preparation of cartilage regenerative scaffolds [24]. Porous collagen/hyaluronic acid matrices have been crosslinked by 1-ethyl-3-(3-dimethyl aminopropyl)carbodiimide (EDC) [25]. Such scaffolds can be used for dermal tissue regeneration. Porous sponges based on the blends of collagen and hyaluronic acid have been fabricated by employing a combination of freezing, lyophilizing, and 1-ethyl-3-(3-dimethylaminopropyl)-carbodiimide (EDC) crosslinking methods [26]. Porous hybrid scaffolds based on collagen and hyaluronic acid have also been prepared by a novel overrun process performed by Lee et al. [27]. The above-mentioned authors showed that the scaffolds with uniform dual-pore structure with porosity higher than scaffolds prepared by the conventional freeze-drying method can be prepared. In this work, the bubble injection and recrystallization have been used. The mechanical strength and biodegradation kinetics were controlled by choosing the adequate preparation method and the collagen/hyaluronic acid composition. The porous collagen/hyaluronic acid scaffolds could be functionalized with biotin by incorporating avidin. A nano-fibrous assembly of collagen–hyaluronic acid for controlling cell-adhesive properties has been studied by Fujie et al. [28]. Hybrid scaffolds composed of hyaluronic acid and collagen in a 3D form were prepared and evaluated for cartilage regeneration by Kim et al. [29]. The time of degradation of the hybrid scaffolds in vitro increased with increasing collagen concentration. Moreover, the cell growth in the hybrid scaffolds increased with increasing collagen concentration after a period of 2 weeks of the cell culture.

Multifunctionalized hydrogel scaffolds based on hyaluronic acid–tyrosine and human-like collagen have been fabricated by Liu et al. [30]. The crosslinking of this blend with 1,4-butanedioldiglycidyl ether led to the formation of materials that can be considered as soft-tissue fillers. It has been shown that the material showed good mechanical properties, biological stability, and biocompatibility.

An extracellular matrix can be mimicked by biopolymer blends in several forms. An injectable hydrogel composed of type I collagen and hyaluronic acid has been designed to mimic the extracellular matrix for vascular cells growing and wound closure by Ying et al. [31]. The preparation of the collagen/hyaluronic acid hydrogel was performed through in situ couplings of phenol moieties of collagen I-hydroxybenzoic acid and hyaluronic acid–tyramine through horseradish peroxidase. The porous hydrogel was obtained using the above procedure that contributed to the exchange of gas, medium, and nutrition. The injectable hydrogel plays an important role in soft-tissue filling and repair. Such an injectable hydrogel based on hyaluronic acid and human-like collagen was proposed by Zhang et al. [32]. Collagen and HA were crosslinked with 1,4-butanediol diglycidyl ether to form a three-dimensional network. An in vivo injection showed that there was little inflammatory response to such a hydrogel after 1, 2, and 4 weeks. This hydrogel can also be considered as a promising biomaterial for soft-tissue filling and repair. Collagen/hyaluronic acids hydrogels can be used as an efficient and controlled gene delivery biodegradable materials [33]. Biomaterial-based gene delivery can find numerous tissue engineering applications or can be a tool to examine tissue formation.

Collagen/hyaluronic acid blends can be used as an artificial dermis [34]. The efficacy of an artificial dermis composed of hyaluronic acid and collagen with or without epidermal growth factor, both in vitro and in vivo, have been studied by Mineo et al. [34]. It was found that such artificial dermis induced excellent wound bed formation acceptable for autologous skin grafting. Collagen/hyaluronic acid blends have been studied as wound dressing by several research groups as well [35,36,37]. A modified collagen/hyaluronic acid mixture has been studied as an injectable composite filler for soft-tissue reconstruction and may be a suitable candidate as an injectable dermal filler for tissue augmentation in humans [38].

Another procedure in the fabrication of new materials based on biopolymer blends is electrospinning. A nanofibrous hyaluronic acid/collagen hybrid scaffold has been fabricated by electrospinning by a number of research groups [39,40]. The two above mentioned substances formulated interpenetrating polymer networks, which have been studied for their implementation as a tissue-engineered heart valve. The review concerning this approach has been prepared by Nazir [41]. In the above-mentioned review, the comparison of the existing experimental approaches and recent technical challenges in this field have been demonstrated.

## 3. Blends of Hyaluronic Acid and Chitosan

Chitosan is a polysaccharide, which is obtained from chitin. It shows cationic polyelectrolyte properties. In chitosan structure, there are 2-acetamido-2-deoxy-β-d-glucopyranose units and the deacetylated form of these units, 2-amino-2-deoxy-β-d-glucopyranose. Chitosan is widely used in biomedical, pharmaceutical, and cosmetic applications. This biopolymer is biocompatible with high charge density. It is also non-toxic and shows good mucoadhesion [42]. Chitosan materials can be modified by the addition of hyaluronic acid to form new materials based on such binary blends. Hyaluronic acid and chitosan can form nanoparticles, hydrogels, microspheres, sponges, and films, all with a wide range of biomedical applications. In the scientific literature, there are several papers regarding an investigation of interactions between chitosan and HA [43,44,45,46,47,48,49,50,51,52,53,54,55,56,57,58,59,60].

**Figure 3:**
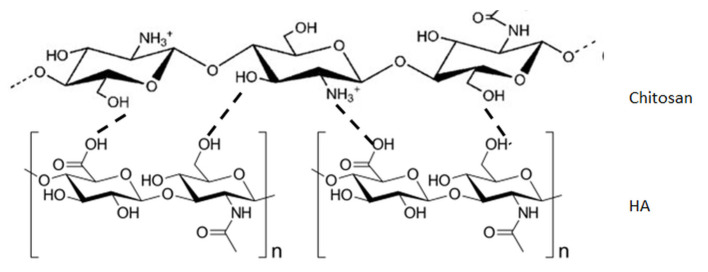
Possible interactions between chitosan and hyaluronic acid (HA) in blends.

The degree of miscibility of chitosan with hyaluronic acid was analyzed by us by a viscometric method and atomic force microscopy [43]. The studies indicated that chitosan/hyaluronic acid blends were miscible at the weight fraction of chitosan w(Chitosan) ≥ 0.5 in 0.1 mol dm^−3^ CH_3_COOH/0.2 mol dm^−3^ NaCl and 0.1 mol dm^−3^ HCl at 25 degrees C. The influence of the type of solvent on the structure of chitosan, hyaluronic acid, and their blend films was investigated as well [44]. It was found that the surface roughness of chitosan, hyaluronic acid and their blended films was altered by mixing. The structure of chitosan blends with hyaluronic acid depends on the blend composition and on the solvent used for preparing the blend. Moreover, very important in miscibility were also the chitosan degree of acetylation and its molecular weight [45]. The possible interactions between chitosan and hyaluronic acid are shown in Figure 3. The interactions between hyaluronic acid and chitosan depend on pH and ionic strength [46]. Due to the strong charge complementarity between both of the biopolymers, electrostatic self-assembly may take place at very acidic pH but is almost unobservable at high ionic strength.

Based on the blends of hyaluronic acid and chitosan the hydrogels have been investigated for applications in tissue engineering to cartilage regeneration [47,48]. The potential of chitosan–hyaluronic acid dialdehyde hydrogels for in vivo cartilage regeneration has been studied as well [50]. In the above-mentioned study, the gel alone for cartilage regeneration as well as a combination of chondrocytes and gel for cartilage repair were studied. It was found that there was no significant enhancement in the quality of regenerated cartilage in the presence of encapsulated chondrocytes. Hyaluronic acid and chitosan have been proposed for the preparation of coacervate-based scaffolds for cartilage tissue engineering [50]. Such coacervates were used also to encapsulate bone marrow stem cells. Injectable and body temperature-sensitive hydrogels based on chitosan and hyaluronic acid for pH-sensitive drug release have been proposed by Zhang et al. [51]. It was shown that the mechanical properties, as well as the gelation temperature, can be modified by changing the HA content. The drug release possibility of such materials can also be modified by the blend composition. It was stated that the carboxyl group in hyaluronic acid can form the hydrogen bonds with the protonated amine in chitosan, which promotes the increase in mechanical strength of the hydrogels and depresses the initial burst release of drugs from the hydrogel. Hyaluronic acid/chitosan polyelectrolyte multilayers were used for surface modification of titanium alloys for biomedical applications [52]. Hyaluronic acid and chitosan-based materials can also be used as a new dressing generation for wound care [53]. Chitosan and hyaluronic acid have been used for the preparation of composite fibers by electrospinning and subsequent coating [54]. The electrospun nanofibers were also obtained from an aqueous complex coacervate solution composed of chitosan and hyaluronic acid [55]. Electrospun nanofibers can be used in biomedical applications. Chitosan and hyaluronic acid have also been studied as a promising biomaterial for injectable tissue engineering as thermogels that undergo temperature-dependent-so-gel transition [56]. Preparation of one pot triple network hydrogel of chitosan and hyaluronic acid formed by triazole linkage, metal-coordination, and polyion complexation was proposed by Engkagul et al. [57]. It was shown that the salt-containing water system favors polyion complex formation of chitosan and hyaluronic acid without precipitation. The mechanical properties and morphologies can be controlled by simply varying the biopolymers mole ratios. The obtained hydrogels showed biocompatibility based on studies with chondrocyte cells. Chitosan–hyaluronic acid polyelectrolyte complex scaffolds were fabricated with statistically significant stiffness variances by Erickson et al. [58] to characterize the effect of scaffold stiffness on morphology, proliferation, drug resistance, and gene expression in human glioblastoma cells. Chitosan–hyaluronic acid blends have also been used for the fabrication of wound-healing materials [59,60]. New materials in the form of membranes composed of chitosan and chitosan–hyaluronic acid containing new arginine derivatives with thiazolidine-4-one scaffold have been prepared using the ionic cross-linking method by Iacob et al. [60]. The properties of chitosan and HA allow for several modifications of both of these biopolymers, so probably in the future, several new materials based on chitosan and HA blends will be proposed.

## 4. Ternary Blends of Hyaluronic Acid, Collagen, and Chitosan

More than two polymers blending is a new trend in polymer science. In the most recently published literature, there are reports on ternary blends prepared using three different polymers. Although it is not easy to investigate the interactions between three different macromolecules, some attempts have been made to understand the chemical and physical forces acting on the molecular level. New materials based on ternary biopolymer blends have also been proposed. There are only a few papers in the scientific literature regarding hyaluronic acid blends with other natural polymers and synthetic polymers where interactions between biopolymers in mixtures have been scarcely described. Nevertheless, the interactions between collagen, hyaluronic acid, and chitosan in mixtures and materials based on collagen/hyaluronic acid/chitosan blend have been studied within the last 5 years [61,62,63,64,65,66,67,68,69,70,71,72,73,74,75,76] and new materials have been proposed.

**Figure 4:**
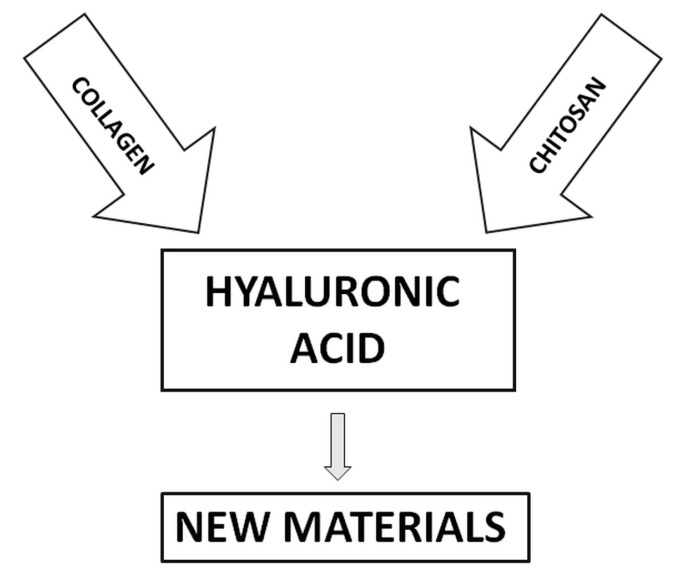
Scheme of preparation of the materials based on hyaluronic acid, collagen, and chitosan.

In Figure 4, the scheme of preparation of the materials based on hyaluronic acid, collagen, and chitosan is shown.

Collagen, hyaluronic acid, and chitosan possess specific properties that can be used to produce man-made blends that confer unique structural and mechanical properties. Miscibility of the components is an important aspect, which determines the properties of the ternary blend. The interaction between three natural polymers, namely collagen, hyaluronic acid, and chitosan, have been studied by viscometric measurements and FTIR spectroscopy [43,62]. The viscometric study showed that collagen/hyaluronic acid blends were miscible in any composition. In the case of ternary blends, the polymeric components were partially miscible. The mechanical properties of chitosan/collagen/hyaluronic acid films, such as tensile strength and Young’s modulus depend on the blend composition. The addition of chitosan to the collagen/hyaluronic acid blend led to an increase in tensile strength by approximately 50%. The results of FTIR analysis showed intermolecular interactions between functional groups of biopolymers [62].

**Figure 5:**
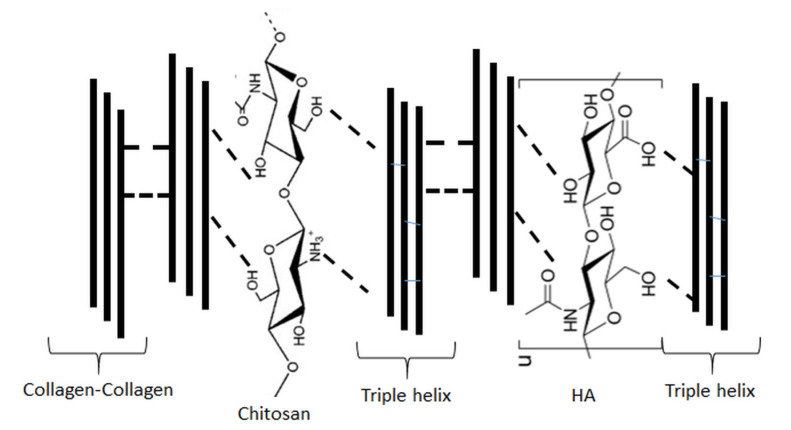
Possible interactions between collagen, chitosan, and hyaluronic acid in blends.

The interactions in chitosan blends with hyaluronic acid and collagen have been studied in solution by the viscometric method and by atomic force microscopy and FTIR for films made of the blends. The surface properties have been measured by contact angle measurements. The results showed that chitosan/hyaluronic acid blends were miscible at the weight fraction of chitosan w(ch) ≥ 0.5. In the case of ternary blends, the polymeric components showed miscibility with collagen at a weight fraction smaller than 0.2. The wettability of chitosan/hyaluronic acid and chitosan/hyaluronic acid/collagen blend films was bigger than those for the chitosan and collagen films. The morphology of ternary blends has also been altered in comparison to films made of single biopolymers. Atomic force microscopy (AFM) results showed that microdomains appeared in the form of globular agglomerates. The differences between surface properties of films made of single components and the surface properties of films made of ternary blends can be a consequence of interactions between collagen, chitosan, and HA [42,63]. The possible interactions between above-mentioned biopolymers have been presented in Figure 5.

Based on chitosan, hyaluronic acid, and collagen blends, thin films and 3D sponges have been prepared [63,64,65,66,67]. The properties of blend films have been investigated by contact angle measurements and atomic force microscopy. The former measurements showed that chitosan films are more polar after the addition of hyaluronic acid and collagen. The AFM results showed that the addition of chitosan to hyaluronic acid led to an increase in surface roughness [64].

**Figure 6:**
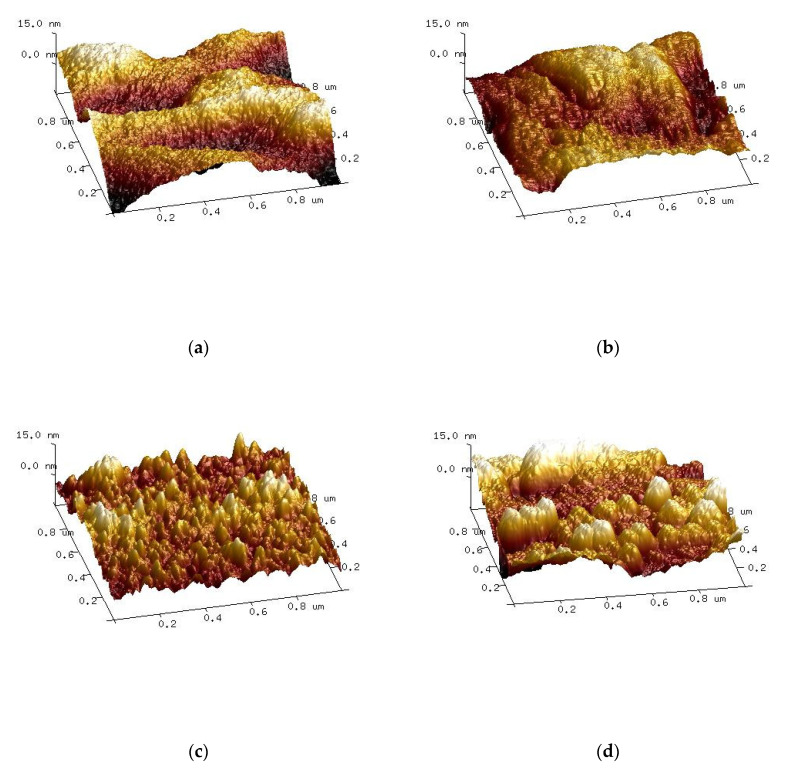
AFM images of the surface of unmodified and samples modified with dialdehyde starch (DAS): (**a**) collagen, (**b**) collagen + DAS, (**c**) chitosan, (**d**) chitosan + DAS. [67], Copyright 2020. Reproduced with permission from Elsevier Ltd. (DAS = dialdehyde starch).

The addition of chitosan to collagen/HA mixture led to the alterations of surface roughness, hydrophilicity, and thermal properties of those binary blends. Thermal stability of binary blends increased after the addition of chitosan. The surface free energy has been also altered after mixing of three biopolymers. Polar and dispersive components of surface free energy, which were calculated for the binary and ternary blends showed that more hydrophilic films were produced by HA and chitosan addition to collagen. All the above observations suggest that collagen interacts with hyaluronic acid and chitosan. The interactions between three macromolecules led to the change in the surface properties of polymer films [65]. Surface properties of ternary blends are very important as biopolymer films made of the ternary blend can be applied for example in cosmetic preparations [66]. The hair protection capability of collagen/chitosan/hyaluronic was studied using SEM microscopy and the mechanical testing of hair coated with the blends. It was found that the addition of hyaluronic acid to a collagen/chitosan blend improves the mechanical resistance of biopolymeric films. Samples with hyaluronic acid addition were more stable in an aqueous environment and provided higher surface roughness. Films based on chitosan, collagen, and hyaluronic acid can be successfully crosslinked by dialdehyde starch [67]. It was found that the addition of dialdehyde starch had an influence on mechanical properties of the films. The films crosslinked with dialdehyde starch were less elastic and more resistant to rupture than those without such treatment. The roughness of the samples decreased after the crosslinking with dialdehyde starch (Figure 6) and the surface free energy increased. The film-forming properties of the mixture of collagen, hyaluronic acid, and chitosan crosslinked with dialdehyde starch can be used in medicine and in cosmetic preparations.

**Figure 7:**
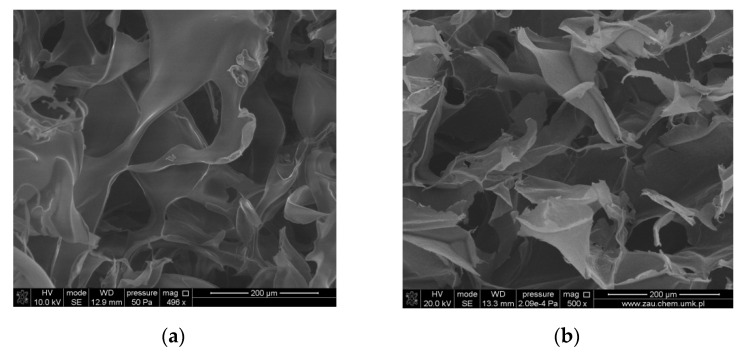
SEM images of (**a**) CTS/Coll; (**b**) CTS/Coll/5HA composites. [68], Copyright 2020. Reproduced with permission from Elsevier Ltd. (CTS = chitosan; Coll = collagen; HA = hyaluronic acid).

3D porous composites based on blends of chitosan, collagen, and hyaluronic acid were obtained through the lyophilization process, and the properties of the scaffolds were studied [68]. SEM images of such 3D composites are shown in Figure 7. Biological properties are also significant, especially for those materials that are dedicated to biomedical application. Natural polymers have been widely used in biomedicine and separately have been proposed as the in vitro extracellular matrix materials. However, the interactions of tricomponent–biopolymer composites with cells is not well studied yet. Biological properties of new materials obtained based on ternary blends should be investigated. For each blend, the proliferation rate of selected cells incubated with biomaterials should be studied. Hyaluronic acid addition to chitosan/collagen blend also modified the properties of 3D composites based on those biopolymers. The results showed that mechanical properties and thermal stability of chitosan/collagen blends were improved. Biological properties of 3D materials can be sufficient for biomedical applications, as it was found that materials were non-toxic and the cell morphology was not significantly altered [68].

Three-dimensional porous polymer-based matrices can be used for fabrication composite materials containing inorganic particles. Such materials have potential in bone repair and in bone tissue engineering. It seems that 3D porous composites based on blends of chitosan, collagen, and hyaluronic acid can also be considered as a matrix for the incorporation of inorganic particles [69,70,71,72,73,74,75,76]. Additional crosslinking of such composites may offer materials with good biocompatibility and mechanical properties, which can be proper in bone tissue engineering. For example, the calcium phosphate in situ precipitation in 3D porous scaffolds based on chitosan, collagen, and hyaluronic acid crosslinked by EDC/NHS (N-(3-dimethylamino propyl)-N’-ethylcarbodiimide hydrochloride/N-hydroxysuccinimide) was proposed [69]. It has been shown that the properties of 3D composites crosslinked by EDC/NHS were altered by hyaluronic acid addition. The following parameters of the scaffold were improved: mechanical properties, thermal stability, and porosity. SEM images showed that precipitation was homogeneously carried out in the whole volume of samples. Attachment of SaOS-2 cells to all the modified materials was more efficient in comparison to results obtained in unmodified control, and proliferation of these cells was markedly increased on scaffolds with precipitated calcium phosphate. The obtained materials can be potentially used in tissue engineering and regenerative medicine. Not only films but also 3D scaffolds based on chitosan, collagen, and hyaluronic acid can be successfully crosslinked by dialdehyde starch [70]. Such crosslinked matrixes can also be used for calcium phosphate in situ precipitation. The mechanical properties, porosity, and density of the materials were improved after such a crosslinking process. Calcium phosphate was deposited in the scaffolds at the Ca/P ratio similar to 2. SEM images showed the homogeneous structure, with interconnected pores. The crosslinker addition and inorganic compound precipitation improved the biocompatibility of the scaffolds. It is much easier and faster is to prepare the polymer/inorganic particles composites by the simple addition of such particles to polymer matrices. It is known that collagen and hydroxyapatite form a complex structure of bone tissue. To produce an artificial bone tissue the powder of nano-hydroxyapatite has been added to the mixture of chitosan, collagen, and hyaluronic acid. After mixing all the above-mentioned components and lyophilization process, the porous 3D composite was obtained. The addition of hydroxyapatite (Hap) caused an improvement of mechanical and thermal properties of ternary biopolymer blend. All the composites showed a porous structure with interconnected pores, which can be appropriate for bone tissue engineering [71,72]. Calcium ions can be released from the composite during its degradation in water [71]. Scaffolds can also be crosslinked by dialdehyde starch and by tannic acid [72]. The compressive modulus, as well as the porosity for the scaffolds crosslinked by dialdehyde starch, was higher than for those crosslinked by tannic acid. However, crosslinking with tannic acid led to material with better biocompatibility than those for materials after crosslinking by dialdehyde starch. Nevertheless, the results showed that both scaffolds can provide the support required in tissue engineering and regenerative medicine [72]. Collagen, chitosan, and hyaluronic acid blends can be also crosslinked with genipin [73]. The above mentioned three biopolymers were used to prepare injectable and in situ gelating biomimetic hybrid materials for potential use in bone tissue engineering. The surface-modified silica particles were introduced to the solutions of biopolymers and after crosslinking with genipin the bioactive phase was formed. Hybrids of various compositions were obtained, and their physicochemical and biological properties were studied. The in vitro cell culture studies showed that the materials developed are biocompatible as they support MG-63 cells adhesion, proliferation as well as alkaline phosphatase (ALP) expression [73].

Although the biological properties in vivo of single natural polymers are widely researched, the behavior and influence of tricomponent–biopolymer composites on cell morphology, differentiation, and function in living organisms are not yet well known. For collagen/chitosan/hyaluronic acid composites, the adhesion and proliferation of human osteosarcoma SaOS-2 cells on the scaffolds have been studied and the biocompatibility of the chosen scaffolds has been further studied by their in vivo implantation into the subcutaneous tissue of rabbits [74]. The obtained results suggest that the stability of such scaffolds is rather low. The X-ray images of the tissues surrounding the scaffolds showed both the control scaffolds without hydroxyapatite (HAp) and those with 50% wt. HAp addition underwent degradation after 6 months. The scaffolds containing 80% wt. of HAp remained in the implanted place, which may suggest that it can be useful in tissue engineering. Biological properties and tissue response show that the material can be considered as a scaffold of soft and hard tissues.

Collagen, chitosan, and hyaluronic acid in the form of thin films can be used as a matrix for drug incorporation [63,75]. Polymeric blends based on the above-mentioned biopolymers in the form of thin films with the addition of gentamicin sulphate were obtained. Microbiological tests were performed to evaluate the diffusion of the drug from matrices. The results showed that thin films based on collagen, chitosan, and hyaluronic acid enriched in gentamicin sulphate inhibit the growth of both Gram-negative bacteria (E. coil and P. aeruginosa) and Gram-positive (*S. aureus*) ones [75]. The procoagulant properties of hyaluronic acid–collagen/chitosan complex film have also been studied [76,77].

Collagen, chitosan, and hyaluronic acid in a 3D form can be used as a matrix for magnetic particles incorporation [78]. It was found that 3D composites made of collagen, chitosan, and hyaluronic acid with magnetic particles are hydrophilic and characterized by a high swelling ability; nevertheless, they are rigid and lack flexibility. With the increasing content of magnetic particles in the polymer blend, the Young’s modulus decreases. The 3D material with magnetite particles can be used in biomedical applications, e.g., tissue repair and drug delivery.

## 5. Possible Application of New Materials Based on the Blends of Hyaluronic Acid with Other Natural Polymers

New materials based on the blends of HA, collagen, and chitosan can be prepared in several forms. Film-forming properties can be useful for the preparation of wound-healing materials [2,63,64,65,66,67,79,80]. Mechanical properties of such materials can be modified by chemical and physical crosslinking [67,72,73,81,82]. Such modification may help in stimulating epithelium cells to proliferate, which are often needed to produce materials with improved biological properties for tissue engineering [81,82]. The properties of the materials based on the blends can be simply modified by changing the weight ratio of the components in the blend. Thin films can be also used in tissue engineering, as usually, materials obtained have been biocompatible.

New materials based on the blends of above-mentioned biopolymers can be used as a topical formulation, which can offer some potential for the delivery and localization of medication to the skin [83]. Some antibacterial properties can be reached by incorporation silver nanoparticles and/or other bioactive agents into formulation [75,84,85]. The penetration through the skin can be adjusted by a properly selected molecular weight of HA [86]. A cosmetic formulation, such as a cream for the treatment of skin disorders and dehydration caused, for example, during radiotherapy and other treatments, may contain a blend of HA, collagen, and chitosan.

Polymer blends can be also turned into 3D structures, such as foams and scaffolds [68,69,70,71,72,87,88,89]. In such 3D structures, inorganic particles can be incorporated, and in such a way, one can obtain materials, which can mimic a bone tissue [72,74]. Incorporation of magnetic particles into the biopolymer blends can lead to new materials, which can be used in biomedical applications, such as: tissue repair, drug delivery, magnetic resonance imaging (MRI), hyperthermia, magnetofection, and cellular therapy [78,90,91,92,93]. 3D scaffolds based on biopolymer blends with magnetic properties can be inserted directly into an injured site. In such a way, it can help to control the orientation of new collagen fibers formed around the applied scaffold. Such new materials can also be used in new generation therapy and as matrices for the delivery of compounds with magnetic properties [78].

Within the last 5 years, several articles have been published that show that it is still of interest within scientific groups to research the blends of hyaluronic acid with other biopolymers [85,94,95,96,97,98,99,100,101,102]. For example, new materials were prepared based on the mixtures of hyaluronic acid and carboxymethyl cellulose [94]. Interpenetrating polymer networks based on collagen, hyaluronic acid, and chondroitin sulfate as scaffolds for brain tissue engineering was studied by Li et al. [95]. Hyaluronic acid–pullulan injectable hydrogels incorporated with biomimetic hydroxyapatite spheres were studied by Ghorbani et al. [96]. Water-insoluble silk fibroin/hyaluronic acid scaffolds were proposed by Guan et al. [97]. Polycaprolactone/gelatin/hyaluronic acid blends have been studied for usage as electrospun scaffolds by Unal et al. [98]. Ternary blends of silk fibroin, hyaluronic acid, and heparin were also studied for potential soft-tissue engineering [99]. A novel biomaterial made of alginate, hyaluronic acid, halloysite nanotube, and polyvinylidene fluoride has been proposed for printing cartilage scaffolds [100]. The hydrogel composed of hyaluronic acid and alginate as a potential bio-ink for 3D bioprinting of articular cartilage engineering constructs has been also proposed [101]. Next potential bio-ink was proposed by enzymatically crosslinked hyaluronic acid–gelatin hybrid hydrogels [102]. There are much more examples of ongoing research on hyaluronic acid and its blends with other biopolymers. This fact suggests that it is increasing interest in the creation of new materials based on such blends. Apart from experimental study on HA materials there are also studies regarding molecular dynamic simulation of this macromolecule behavior in the presence of water and other molecules [103,104].

**Figure 8:**
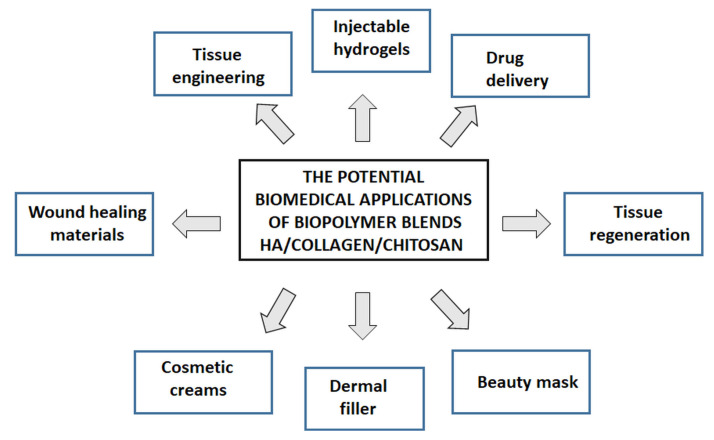
Potential applications of biopolymer blends base of HA, collagen, and chitosan.

The application of biopolymer blends for preparation of biomedical materials has been summarized graphically in Figure 8.

## 6. Conclusions

Hyaluronic acid is widely studied for its use in biomedical fields. This biopolymer can be modified in several ways, especially blending it with another biopolymer may lead to new interesting biomaterials. The preparation of a hyaluronic acid blend with other polymers and/or biopolymers is neither a closed nor a completed topic, as there are many polymers and biopolymers that can be studied as a component of such the blends. In regenerative medicine, there is still an increasing need for new materials for cell-based transplantation, tissue engineering, drug delivery, and gene therapy. There is also a need to design new wound dressing materials and hydrogels for cosmetic applications. New materials based on the blends of hyaluronic acid with collagen, chitosan as well as with other biopolymers may fulfil the gap between the demand for biomaterials for tissue regeneration and the supply. The potential of newly designed materials using biopolymer blends may be huge; nevertheless, the detailed biological study of any kind of such materials is required.
